# Resilient Biodiversity Conservation: Working with Social–Ecological Connections to Navigate Crises

**DOI:** 10.1093/biosci/biaf156

**Published:** 2025-10-23

**Authors:** Erik Andersson, Romina Martin, Pippin Anderson, Shirley Brooks, Gonzalo Cortés Capano, Alberto González-García, Viola Hakkarainen, Marion Jay, Sandra Lavorel, Margot Neyret, Tobias Plieninger, Christopher M Raymond

**Affiliations:** Faculty of Biological and Environmental Sciences, Ecosystems and Environment Research Programme, Helsinki Institute of Sustainability Science, University of Helsinki, Helsinki, Finland; Stockholm Resilience Centre, Stockholm University, Stockholm, Sweden; Stockholm Resilience Centre, Stockholm University, Stockholm, Sweden; Department of Environmental and Geographical Science, University of Cape Town, Cape Town, South Africa; Department of Geography, Environmental Studies, and Tourism, University of Western Cape, Bellville, South Africa; Department of Agricultural, Economics, and Rural Development, University of Göttingen Göttingen, and Faculty of Organic Agricultural Sciences at the University of Kassel, Kassel, Germany; Laboratoire d’Ecologie Alpine, Université Grenoble Alpes, Université Savoie Mont Blanc, Grenoble, France; Faculty of Biological and Environmental Sciences, Ecosystems and Environment Research Programme, Helsinki Institute of Sustainability Science, University of Helsinki, Helsinki, Finland; Department of Agricultural, Economics, and Rural Development, University of Göttingen Göttingen, and Faculty of Organic Agricultural Sciences at the University of Kassel, Kassel, Germany; Laboratoire d’Ecologie Alpine, Université Grenoble Alpes, Université Savoie Mont Blanc, Grenoble, France; Laboratoire d’Ecologie Alpine, Université Grenoble Alpes, Université Savoie Mont Blanc, Grenoble, France; Department of Agricultural, Economics, and Rural Development, University of Göttingen Göttingen, and Faculty of Organic Agricultural Sciences at the University of Kassel, Kassel, Germany; Faculty of Biological and Environmental Sciences, Ecosystems and Environment Research Programme, Helsinki Institute of Sustainability Science, University of Helsinki, Helsinki, Finland; Faculty of Agriculture and Forestry, Department of Economics and Management, University of Helsinki, Helsinki, Finland

**Keywords:** biodiversity conservation, crisis management, cross-scale dynamics, disruptions, social–ecological systems

## Abstract

Biodiversity conservation needs to adjust and keep adjusting to changing conditions. This is largely a matter of connections—across land uses, between people and the landscapes they inhabit, and between sectors and governance levels. Connections play an important role in shaping landscape dynamics and in the ability of conservation practitioners to be able to draw on resources outside their often limited mandates or authority. Focusing on disruptions, in this study, we discuss the current understanding of three interlinked aspects of conservation where active work with building and strengthening connections can help make recovery easier: landscape cohesion, societal appreciation and support for conservation, and the ability to rewire collaborations and bridge organizational and administrative boundaries. Specifically, we highlight how emerging insights on temporal shifts in connections, from spatial ecology to environmental psychology and crisis preparedness, inform and outline a research agenda for better situating conservation in complex landscapes undergoing frequent changes and disruptions.

The current context of rapid and often unpredictable environmental and social change raises major unanswered questions about how to preserve biodiversity while social–ecological interdependencies are put under pressure. Scholars have noted that modes of biodiversity conservation that could be viewed as effective or efficient under business-as-usual circumstances may not be able to effectively navigate times of crisis and sudden change (Cumming et al. 2015, Colloff et al. 2017, Scoones 2024). In response, more recent conservation policies have initiated a shift toward a broadening of conservation efforts. For example, the Kunming–Montreal Global Biodiversity Framework raises the importance of making land and seas outside formal biodiversity conservation part of the overall conservation effort (CBD 2022) through “other effective conservation measures” (CBD 2018). It also advocates for “well-connected and equitably governed systems of protected areas and other effective area-based conservation measures… [to be] integrated into wider landscapes” (CBD 2022, p. 9). However, even this expanded and inclusive understanding of biodiversity conservation remains unaware of the added pressures from more frequent social–ecological disruption and crises.

Alongside Selman (2002), Hodgetts (2018), and Kremen and Merenlender (2018), in the present article, we use a social–ecological perspective and position *connections* as a multifaceted, cross-cutting lens for analyzing conservation practice and outcomes. We argue that adapting conservation strategies to contemporary challenges will require more attention to critical social–ecological connections and to the ways positive conservation outcomes (both increased biodiversity and stronger, more meaningful connections between people and nature) emerge from interacting entities (box 1). Thinking in terms of connections sheds new light on the spread of and response to disruptions (box 1).

Box 1. Terminology.

Multiple terms are used to capture similar things within and across different fields of research—terms such as *connections, connectivity, relations, relationships, bonds, continuity, interdependency*, and *coherence* among them. In the present article, we take *connections* to connote the exchange between two or more entities and the qualities, structures, and circumstances that allow for this interaction. We understand *relations* or *relationships* as involving people and also including emotional or ideological positioning of oneself relative to others or to nonhuman entities.

Similarly, *crisis, disturbance*, and *disruption* are all used as systemic concepts referring to radical changes in the operation of various types of systems from governance to activities, habits, and values to landscape configurations and dynamics. We primarily use *disruption* to connote an event or a trigger and crisis as the outcome. That term is often used for social–ecological systems, whereas *disturbance* is used in relation to biophysical systems. The two are not always easy to separate.

The importance of ecological connectivity is well established in the biodiversity conservation literature, not least because it enables biodiversity to respond to changing circumstances (e.g., tracking a climatic niche as it moves or recolonizing a site after a disturbance; Hansen and DeFries 2007, McGuire et al. 2016, Fahrig 2017). Biodiversity conservation is also a set of socioculturally embedded practices informed by and sensitive to spatial, temporal, and relational connections. As such, biodiversity and its conservation are also something people relate or connect to in many ways—emotionally, cognitively, and in practice (Hannah 2008, Ives et al. 2018, Raymond et al. 2023). Furthermore, with ecosystem dynamics that span the boundaries used in formal biodiversity conservation, land use on the lands surrounding protected areas can be decisive for the functioning and quality of the protected areas (Hansen and DeFries 2007). Finally, yet other connections are critical for mobility and accessibility, transfer of resources or knowledge, and organization of collaborative governance and collective action (e.g., Bodin 2017, Locatelli et al. 2025).

Biodiversity conservation must adjust and keep adjusting to changing conditions (Colloff et al. 2017). We posit in the present article that producing or coproducing and governing systemic ecological, social, cultural, organizational–institutional, and economic conditions is essential for achieving biodiversity conservation. Certain conditions allow for key connections across complex landscapes to be more easily managed and employed to tap into the different resources needed for conservation. We further contend that attention to ecological, personal, and social connections is especially important during and after crises and disruptions, from local events (e.g., pathogen outbreaks or collapsing local populations) to regional ones (e.g., resistance from marginalized communities to protected areas or novel uses of natural resources) and to broader-scale and higher institutional level issues (conflict and trauma, conflicting policies, or targets and global environmental change; e.g., Cumming et al. 2015, O’Brien et al. 2023).

Using disruptions as the lens, we raise and discuss scalar or cross-boundary dynamics and consider how certain conditions and connections enable different landscape dynamics and support biodiversity-supportive responses. With this as the entry point, we start with the theoretical reasoning behind our analysis, before unpacking and discussing the interrelations among people, nature, and land uses as described in and emerging from the literature.

## Theoretical framework: The role of cross scale links and support for recovering from disruptions

Connections, including in governance, play an important role in shaping spatial dynamics and changes in connections and relations can both trigger change and shape the responses to it. In 2002, Gunderson and Holling launched the idea of panarchical system dynamics (Gundersson and Holling 2002). Their thinking was based on ecological theory and empirical evidence, but the hypothesis was that the described dynamics could be found in social–ecological systems in general. Central to the idea was that connections across spatial scales and boundaries could serve as a unifying lens for understanding landscape dynamics. Panarchy theory is focused on the spread of disturbances and the responses to it. Both disturbances and responses are positioned as multiscale issues; disturbances (*revolt*) scale up and cascade through systems, whereas responses, especially reorganization (*remember*), are informed by resources or assets available at larger spatial scales. *Revolt* is especially likely when adjacent areas are not only connected in terms of flows and dynamics but also connected in their species composition and successional stage. The notion of remembering—recovery or reorganization—is based on connections to and inflow from external, intact species communities or populations, as has been evidenced and described in detail through, for example, recolonization within metapopulation dynamics (Hanski 1999). However, the role of connectivity is not constant over time; a certain degree of dissimilarity and, therefore, reduced connectivity is important for maintaining diversity (Loreau et al. 2003) and for limiting the spread of natural disturbances (e.g., fire, windthrow) and harmful biota (e.g., weeds, pathogens, invasive alien species), which suggests that a higher degree of connectivity would be desirable after a disruption but not before.

The extension of panarchy theory to social–ecological systems remains an open field of exploration. In the present article, we point to two additional aspects of panarchical behavior: First, studies show how wider societal appreciation and support is key for conservation. Like the large-scale ecological memory described by the original panarchy theory, the probiodiversity values held by society can reinforce conservation efforts (e.g., Ives and Kendal 2014, Raymond et al. 2022, Soga and Gaston 2024). Conversely, the loss of emotional and cognitive connection could negatively affect conservation outcomes, as was exemplified by Brom and colleagues (2020), who demonstrated that poor or weak connection resulted in a lack of proenvironmental sentiment. Such connections at the individual level could play out for example in allocating funding or prioritizing government action. Maintaining meaningful relations that support both human and nonhuman well-being will need to include active handling of, for example, place disruptions (Manzo et al. 2023), traumatic experiences (Della Bosca et al. 2020), and extinction of experience (Soga and Gaston 2016), along with variable ecological and recreational literacy (McBride et al. 2013). Furthermore, interrogating the way nature connectedness informs how people, often located far away from conservation sites, understand and empathize with biodiversity may provide an entry point for understanding societal responses to place disruptions and localized crises. Although connections in this sense are held to be positive before, during, and after a disruption, the nature of the connections may need to change to effectively inform responses to the disruption.

Second, similar to the ability to connect across scales—emphasized by panarchy theory—the ability to bridge organizational and administrative boundaries has been put forward as key to managing complex issues such as multifunctional land use and cross-sectoral integration (IPBES 2024). However, finding commonalities, sharing resources, and aligning actions across sectoral or administrative boundaries are only one aspect of effectively managing boundaries (van Broekhoven et al. 2015). In the present article, fields such as organizational studies have helped build an understanding of the construction and evolution of boundaries and interactions across these as complex, socially constructed, and negotiated entities to understand organizational change and multiactor interaction (e.g., Heracleous 2004, Santos and Eisenhardt 2009, Mørk et al. 2012). The ability to reach out to and engage other sectors and other actors, mobilize diverse resources and activate alternative governance modes can be seen as another layer of the *remember* function of a panarchy. Trade-offs between high involvement and heterogeneous governance coalitions and efficiency suggest that more effectively switching back and forth between different modes may be a better fit with variable circumstances (e.g., Boin et al. 2014, Berthod et al. 2017, Ansell et al. 2023).

## Conservation, crisis, and shifting connections

What, then are the critical connections that may help ensure sustained continued conservation efforts through disruptions and crises? The following sections outline both well-established, although often disconnected knowledge and new, tentative insights on how connections are key to navigating biodiversity conservation through crises.

### Supporting diverse biodiversity refugia and recolonization in a dynamic landscape

Biodiversity conservation has different priorities depending on circumstances—from maintaining heterogeneity and distinct differences within gene pools and containing disturbances to connecting disaggregated resources and facilitating recolonization and extending the ecosystem functional area, and these different priorities call for more or less connected landscapes. Landscape ecology has long recognized landscapes as complex and dynamic systems, with key processes such as ecological succession, land-use change, shifting community composition, phenological shifts, and disturbances (e.g., Turner and Gardner 2015, Allen and Starr 2017). Even under normal circumstances, protected areas require supporting structures beyond their boundaries if key ecological functions such as complementary wintering areas, supplementary food sources, or hydrological functioning are to be maintained (e.g., Grumbine 1994, Hansen and DeFries 2007, Mathevet et al. 2016) and, likewise, if the protected areas are to function as biodiversity source areas that support higher biodiversity in adjacent land uses (e.g., Hansen 2011, Brodie et al. 2012). Changes in habitat fragmentation, land management practices, and the assemblages of species within and outside protected area networks may all have profound impacts on landscape connectivity (e.g., Crooks and Sanjayan 2006, Symes et al. 2016). The consequences may become even more apparent in the light of climate and other environmental changes when plants and animals need to adapt by tracking and moving with suitable environmental conditions (e.g., McGuire et al. 2016, Costanza and Terando 2019), shift to new migratory routes (Kauffman et al. 2021), replenish failing metapopulations (e.g., Schlägel et al. 2020), or track variable resources across space (Abrahms et al. 2021).

**Figure 1.**
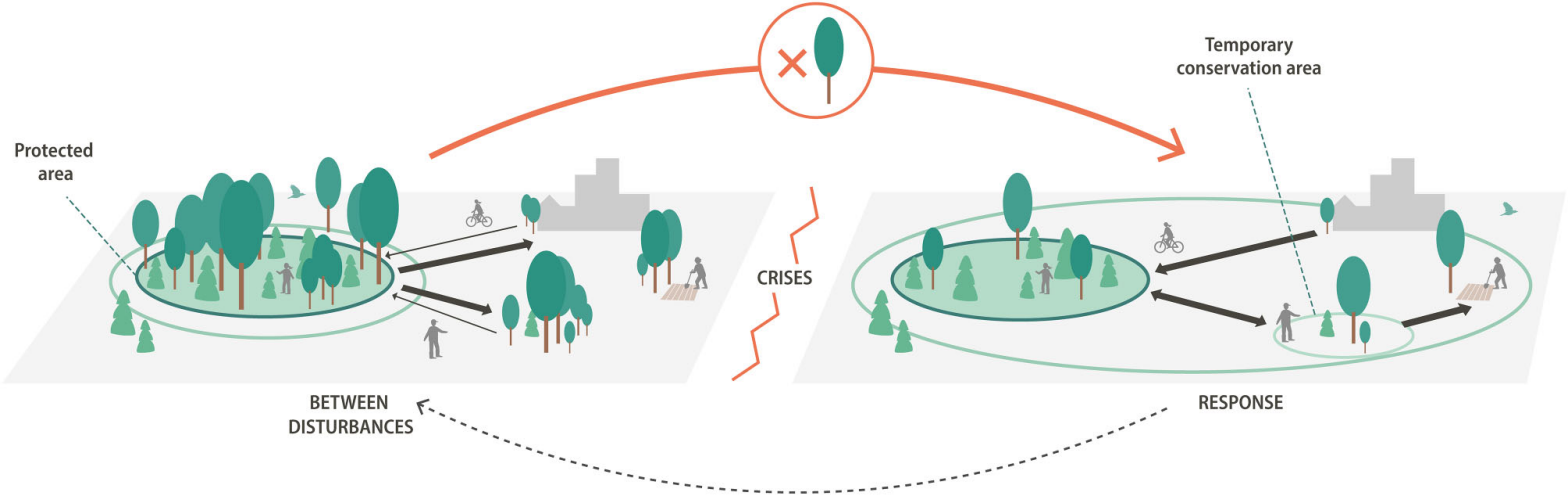
Changing landscape dynamics in responses to crises. Crises and disruptions may rescale or reconfigure the functional conservation area (the thin ring outside the protected area) and the nature and direction of ecological flows across the landscape. When the landscape is disturbed, conservation outcomes become more dependent on the larger landscape surrounding protected areas and exchange between areas. More resources and effort may need to be invested in unprotected land—for example, by assigning land as temporary protected areas (illustrated by the smaller, light green circle). Eventually, as the landscape recovers, land use could return to something similar to the precrisis configuration. The red and black arrows indicate the timeline of a disturbance cycle. (Graphic design: E. Wikander/Azote)

Conversely, protected areas have traditionally been expected to function as biodiversity refugia, with the potential to also function as sources ensuring higher biodiversity in adjacent land uses (e.g., Hansen 2011, Brodie et al. 2012). Moreover, refugia, or resources for coping and recovery over time, may move around in a landscape depending on circumstances (Bengtsson et al. 2003). This means that different locations and land uses may change in their roles within conservation and that places falling outside formal conservation may become critically important during certain times or circumstances (figure 1; e.g., Anderson et al. 2023). Similarly, opportunities for movement may exist only during limited windows of time as conditions change (Zeigler and Fagan 2014). Despite the recognition of complementarity, fluctuating habitat quality and ongoing change, these dynamics remain poorly integrated into conservation strategies, especially over seasonal, annual, and decadal timescales (Zeller et al. 2020, Jones et al. 2025). In all, this makes it difficult for individual protected areas or protected area networks with fixed geographical locations and boundaries to respond to the dynamics of global to local change (e.g., Araújo et al. 2011, Hoffmann et al. 2019). Shifting conditions call for easier switching between alternative landscape level management arrangements and land-use designations (e.g., Fuller et al. 2010, Alagador et al. 2014, Prober et al. 2019). For example, a forest originally managed for timber extraction or carbon sequestration might need to transition into an agropastoral system to mitigate risks of fire ignition and spread (Wolpert et al. 2022). Similarly, changing crop rotations or forest harvesting age can change the ecological dynamics of a landscape and allow for different functional connections (e.g., Tscharntke et al. 2012, Burel et al. 2013). The relative ease of such adjustments may depend on history, the current successional series of neighboring areas, and the length and nature of management cycles (e.g., Foster et al. 2003, Kleyer et al. 2007). Looking to populations rather than land uses, new circumstances may call for rethinking potential conservation assets. For example, the viable population of the frog *Discoglossus galganoi* in an urban park in Madrid could become a conservation asset if the species’ conservation status becomes more critical in the wider countryside (Sánchez et al. 2015). Making more active use of such refugia may take additional efforts, such as mediated translocation, as is evident in the case of the western leopard toad, *Amietophrynus pantherinus*, which needs the efforts of dedicated local conservationists and city authorities outside formal conservation areas in the City of Cape Town to assist it in safely crossing roads in order to access wetlands for mating (Marais-Potgieter and Faraday 2022).

#### Emergent insights

 A certain degree of fragmentation at the landscape level is a prerequisite for response diversity and differential impact of disturbances (e.g., Lavorel 1999, Berkes and Folke 2002, Schippers et al. 2015), and maintaining contrast and ecological discontinuities is important to reduce the risk of widespread disruptions. Conversely, a more connected landscape supports dispersal and redistribution of genetic material after a disruption. Recent studies have started to look into the role of and potential opportunities in temporal variability in connectivity (e.g., Uroy et al. 2021, Marco Palamara et al. 2023). Advances in this area may help fine-tune conservation strategies to better fit local conditions and variable times.

Changing the shape, area, or buffer zones of protected areas to, at least temporarily, include new areas for conservation may provide habitat substitutes or conduits for recolonization after a disruption or disturbance. For example, a viable species population in a nonprotected area could become a conservation focus if the species’ conservation status becomes more critical, locally or overall (e.g., Heywood 2019).

Successional stages, crop cycles, and other time-based processes can influence connectivity (Kleyer et al. 2007). For example, a forest planted for harvest might require a delay in logging or a shift in management—such as increasing species diversity—if it becomes crucial for connectivity with regenerating areas after a nearby fire. Similarly, disruptions affecting certain species may require adjusting crop rotations or fallow periods to meet new conservation needs (Burel et al. 2013), and temporary corridors could link up the landscape when needed (Van Langevelde et al. 2017).

### Personal relations to nature and their relevance for dealing with crisis

Active management is often required to ensure successful conservation of biodiversity and other assets and benefits (see, e.g., Andrade and Rhodes 2012, Vlami et al. 2017). The necessary investment means that biodiversity conservation depends on strong support from the general public, especially in times of disruption and crisis when several issues compete for limited attention (Hausmann et al. 2025) and when more and likely different resources, usually external to the protected areas, need to be mobilized (e.g., Young and Castro 2021, Hermoso et al. 2022). Applied to the context of protected areas, such studies reveal how people–nature connections shape conservation and how protected areas can enable or hinder people–nature connections. For instance, Indigenous connections to land and Indigenous and local knowledge can support biodiversity conservation (Burke et al. 2023) and inform ecosystem restoration (Sena et al. 2022). Biocultural restoration approaches, based on the connections of local communities to nature—the knowledge, uses, and cultural meanings they attach to their territories—showcase how connections to nature can promote benefits and recovery for people and nature after disturbances such as wildfires (Pereira et al. 2023). In urban settings, connections of the people to urban nature, including access and physical experiences, appreciation of beauty, natural vegetation and biodiversity richness, or cultural meanings of urban green areas, affect well-being and social cohesion in urban communities (Clarke et al. 2023).

There is less work on how humans’ relationships with biodiversity inform and are informed by disruptions and crises. Just as felt connectedness may motivate mobilization of resources and other efforts to mitigate or recover from disruptions, as is suggested by the panarchy perspective, the experience of crisis and trauma may also change the way nature connections are built and maintained (Luque-Lora 2024). The COVID-19 pandemic serves as an example of how a social crisis forced people to reassess their available spaces and create opportunities for engaging in different activities or gain access to certain experiences, realigning with both restrictions and opportunities for visiting nature parks and protected areas (Korpilo et al. 2021). Particularly, urban parks saw significant increases in visits, because, in many cases, they were exempted from lockdown policies, and these visits to nature contributed significantly to individual stress relief and mental health recovery (Grima et al. 2020, Geng et al. 2021). Identifying open space locations and visitation policies that ensure accessibility also in the middle of ongoing crises remains a crucial task in the postpandemic response for future park planning (Zhao et al. 2023). Likewise, issues of unequal access to green spaces gain importance in a context of crisis, revealing patterns of environmental justice exacerbated by COVID-19 policies (Gao et al. 2023). This potential marginalization of the ways certain groups connect to nature influences the production and reproduction of different conservation narratives, affecting the ways problems and potential solutions are framed (Wyborn et al. 2020). Similar to urban settings, the importance of forests as critical infrastructure for people’s well-being was amplified during the pandemic (Derks et al. 2020). Visitor patterns (duration and type of activities) in forests changed significantly and demonstrated the restorative effects on psychophysical health and community well-being (Ciesielski et al. 2023, Wunderlich et al. 2024). Therefore, novel situations or crises may call for temporary changes in the regulations set down to guide the use of both protected and unprotected areas. For example, temporary adaptations in visitor numbers for protected areas may call for spatially segregated approaches to open up and restrict access within the same time scale.

**Figure 2.**
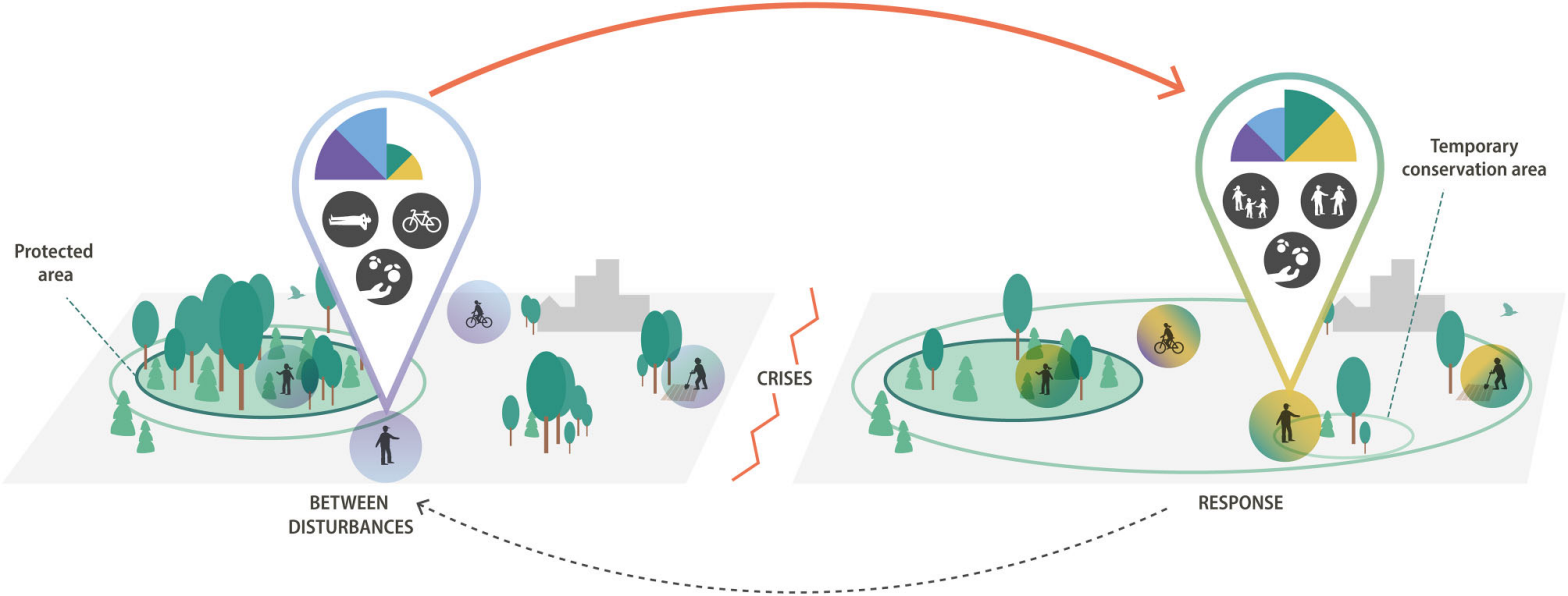
Crises and people–nature connections. Personal values (the pie tiles), needs (the color of the halo around the actors) and activities (the black and white symbols) in human–nature relationships are likely to be different during or just after and between crisis events. Crises change both the type of proconservation actions that are needed and where they are needed in larger, mixed protected, and nonprotected landscapes, as is indicated by the green ring outside the protected area, the functional conservation area. This has implications for how actors respond to the crisis and the role they may play in recovery and reorganization. The red and black arrows indicate the timeline of a disturbance cycle. (Graphic design: E. Wikander/Azote)

Crisis and experienced trauma also affect, directly or indirectly, nature connectedness. The Australian wildfires exemplify how a natural crisis may trigger both empathy with nature under pressure and solastalgia, individual distress caused by the degradation of one’s home environment (Galway et al. 2019, Albrecht 2020, Della Bosca et al. 2020). Survivors reported on environmental cues to prepare for fire risk and the experience of frustration, posttraumatic stress, and anxiety when being confronted with fire and later landscapes devoid of the creatures that once lived there (Stanley et al. 2024). The relationships people develop with their landscape are shaped by the full suite of experiences, from the predisruption, through disruption, to postdisruption. The need to mediate the effects of this experience of traumatic landscape change has so far received relatively little attention (Butler et al. 2018). Experienced trauma, understood more broadly, may affect individuals’ ability to relate to and derive well-being outcomes from time spent in nature and their willingness to actively rehabilitate relations to nature (figure 2; Evans 2023).

The capacity of both individuals and their environments to interact in ways that support mutual well-being needs greater recognition as existential connectivity. Individuals engage in behaviors that help them to navigate their way to the resources they need to sustain their well-being only when their social ecology (formal and informal social networks) has the capacity to provide resources in ways that are culturally meaningful (Ungar 2013).

#### Emergent insights

Experiences and relationships from coproducing ecosystem services, motivated by plural values, not only reveal multiple purposes for nature conservation; they also maintain conservation values in nonprotected lands and may need support to not fall between the cracks and to overcome value conflicts around conservation goals (Chapman et al. 2019, CBD 2022). Various often unsubsidized actions such as organic farming efforts for supporting pollinators, conservation of wetlands, or soil improvements with biochar, offer entry points for short term amplification of supporting actions outside formally protected areas. Promoting these efforts could entail recognition and support for other area-based conservation measures (Alves-Pinto et al. 2021), reinforcing positive feedback loops of connections across spatial scales.

Crisis and disturbances reshape the multidimensional configuration of connections to nature, involving changes in emotions, experiences, materialities, and different expressions or recognitions of knowledge, beliefs, and visions (Walsh 2003). Perceived attacks, especially from the outside, on the identities and livelihoods of the managers of unprotected lands—farmers, foresters, pastoralists, hunters, anglers, and others—often lead to a reductionist defense of production values, overshadowing nonproduction values and potentially polarizing conflicts, which may, in turn, undermine on-the-ground activities in support of biodiversity (Elands and Praestholm 2008, Mack et al. 2023). Crisis might instead emphasize tangible and intangible uses and values that reveal—and potentially strengthen—hitherto underperceived connections beyond production and conservation dichotomies.

Exposure is not enough; nature connectedness and proenvironmental behavior are necessary but not sufficient outcomes of people’s exposure to and experience of nature (e.g., Brom et al. 2020). This is revealed not least by the many recent and currently ongoing crises that clearly show how positive attitudes and well-being outcomes (presumed precursors to proenvironmental behavior) benefit from—and sometimes depend on—intermediation (Evans 2023). This indicates a need to look at the complex of experiences together with enabling capacities and situations that allow individuals to build meaningful connections to biodiversity and nature in general and how these intersect with the framings and narratives that are culturally relevant (dominant, marginalized, alternative narratives) in particular contexts (e.g., Bridgewater and Rotherham 2019, Staddon et al. 2023).

### Alternative modes of collaborative governance

Conservation actions and outcomes (especially at larger scales) emerge from the interplay of complex landscapes and nested layers of conservation-relevant policies and practices across multiple sectors (e.g., Nowell 2010, Buijs et al. 2024, Patterson et al. 2024). The relationships between actors, issues, scales, and places become key factors where connections among people and organizations shapes biodiversity outcomes and determines how governance may respond to crisis. These connections can therefore be conceptualized as a repertoire for adaptively managing boundaries and cross boundary interactions via bridging organizations aiming for institutional fitness, as, for example, in catchment-based freshwater management schemes, which supply different capacities and resources when needed (Moore et al. 2024). Conversely, the implementation of biodiversity targets and policy goals is frequently hindered by conflicting governance priorities, limited resources, and poorly coordinated institutional and regulatory frameworks (e.g., Cardona Santos et al. 2023). Adding variability and uncertainty to what might be the relevant ecological scales for effective conservation further emphasizes the need to move nimbly between different contexts and processes for decision-making. Incompatible interests, institutional settings, and rapid, emergent crises make coordination especially difficult (e.g., Cowell and Martin 2003, Derkzen et al. 2009, Prager 2015).

**Figure 3.**
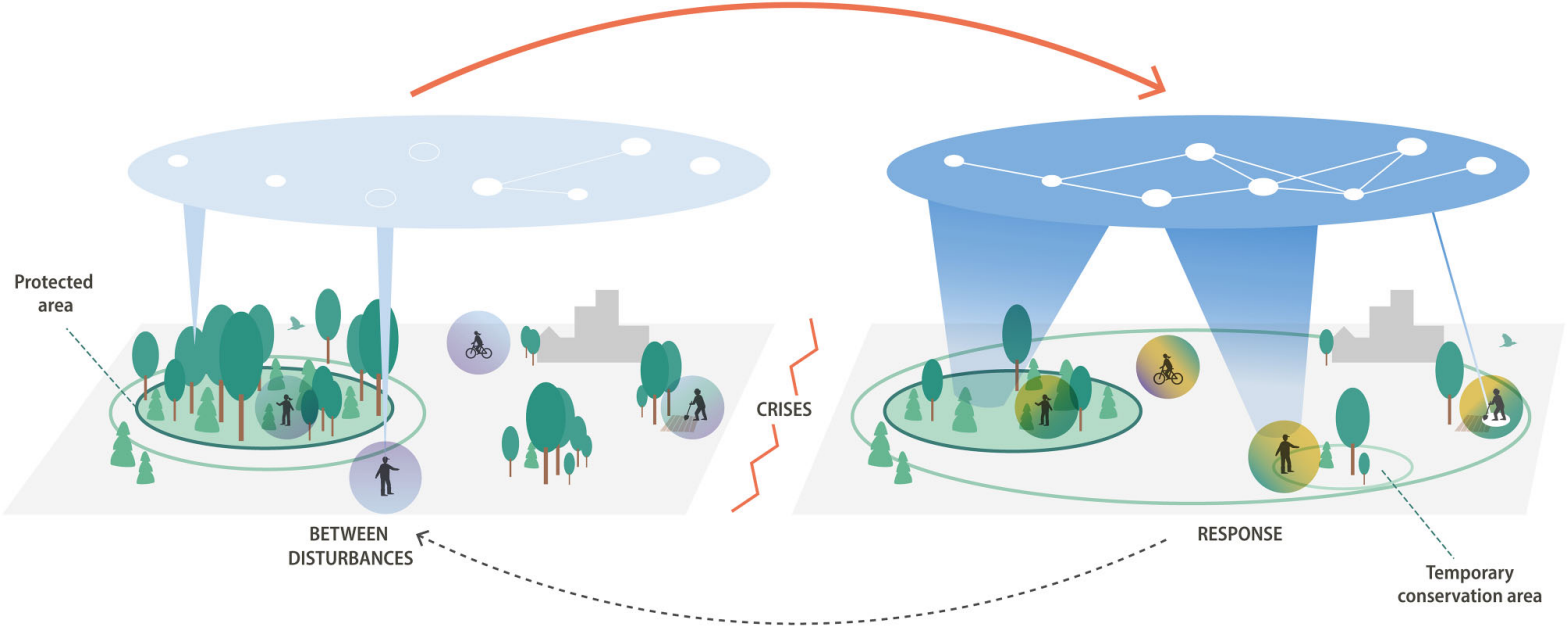
Changes in governance configurations and collaborations in response to crises. Some dormant or new platforms and actors become more relevant and are activated and linked to the response to crises across governance scales (the white nodes and links in the institutional level above the landscape). The effectiveness and capacity of the response and the governance arrangement would be enhanced by a comprehensive understanding of both how to adjust land uses and management and more external resources and backing for actively engaging with and supporting both action and maintained positive relations to nature, especially if informed by an awareness of the diverse human–nature relationships as characterized by plural, changing values and different mandates (the vertical cones; the width indicates the intensity and range of actions and interactions). The red and black arrows indicate the timeline of a disturbance cycle. (Graphic design: E. Wikander/Azote).

New forms of governance based on learning, experimentation, and anticipation are central to addressing biodiversity loss (e.g., Armitage 2008, Muñoz-Erickson et al. 2016, Tanguay et al. 2023). Although conservation is a long-term endeavor, continuity and consistency need to be balanced with flexible, situation-specific strategies that help maintain or mobilize meaningful, reciprocal relations between people and other species. More agile arrangements include regularly monitoring and reevaluating protected areas in terms of not only biodiversity trends and changes but also the way attitudes, uses, and meanings associated with the protected areas change, especially when people or protected areas are under pressure (figure 3; see, e.g., Pahl-Wostl 2009). Managing these relationships requires aligning sectors and often divergent goals, and adjusting structures and processes that are not traditionally seen as part of biodiversity conservation (as has been argued by, e.g., Cumming 2016). This may include posttrauma rehabilitation and education (Evans 2023), and crisis management (e.g., Moynihan 2009, McCumber and King 2020, Thurstan et al. 2021).

Collaboration in governance benefits from being grounded in ongoing deliberation among plural interests, knowledges, and values, and in recognizing power dynamics and politics rather than following siloed, centralized approaches to conservation (Salomon et al. 2023). This includes adopting integrative, inclusive, adaptive, transdisciplinary, and anticipatory governance modes (Visseren-Hamakers et al. 2021). Of course, collaborative governance modes increase complexity as partnerships between multiple actors need to be carefully coordinated and maintained (Waylen et al. 2023). The capacity to promote, accommodate, and ensure inclusion and contribution of local communities to biodiversity conservation and reconciling diverse forms of knowing is well recognized to often lead to better conservation outcomes (e.g., Berkes 2004, Dawson et al. 2021). In times of rapid biodiversity loss and frequent disruptions, governance must also address the social and cultural aspects of recovery and the crucial role of community engagement. For example, following major natural disturbances (e.g., the Australian fires in 2020), protected areas often need ecological connections to source ecosystems for species recovery (e.g., Hansen and DeFries 2007). But at such moments, physical access may need to be restricted to reduce risk and facilitate ecological recovery. Meanwhile, sustaining nonmaterial cultural ties and effective governance during such times is essential for both human and ecological resilience.

#### Emergent insights

Institutional memory for how to organize or reorganize collaborative efforts to deal with disruptions is key for the capacity of actors (individuals as well as organizations) to handle change and emergencies (e.g., Rice and Jahn 2020). Redefining the purposes of a landscape and its patchwork of land uses and practices requires flexibility and adaptive capacity. Different types of circumstances call for new actor constellations, likely at other scales or levels than before the disruption, to be able to mobilize the resources needed to deal with the situation and temporarily restructure conservation efforts (e.g., Jepson et al. 2011, Wyborn 2015, Chaffin and Gunderson 2016).

Short-term changes in mandates and decision-making and permission processes (such as expanded decision-making rights to operational actors, temporary resource allocations) are an emergency response that could be relevant also for biodiversity conservation (see, e.g., Moynihan 2009, Scoones 2024). Existing approaches to zonation and temporarily limited conservation status (compensation payments included) could potentially be expanded to better accommodate short-term strategies for coping with or recovering from disruptions.

Making sense of and responding to crises may be subject to domination by sector-specific interests, the reinforcing of power imbalances and prioritizing the short term over the long term (e.g., Pahl-Wostl et al. 2023, Cortés-Capano et al. 2025). These are undesirable outcomes from a conservation perspective. Current work on multiple preparedness strategies provides new challenges and opportunities (e.g., Sellberg et al. 2024). If done carefully, approaches such as nature-based solutions could help make new connections between biodiversity conservation and other sectors. Strengthening the role of protected areas as resilience pivots (sensu Rotarangi and Stephenson 2014), not only for biodiversity but also for human well-being may help mainstream conservation practice.

## Conclusions

The impact of disruptions, and the role played by connections in dealing with them, is something conservation practice will have to recognize. Connections are not static, and the role of and need for different connections is more variable than currently accounted for in conservation policy and practice. On the basis of the findings in this article, we make three propositions for further research and, tentatively, conservation governance:

First, guidance for when to invest in protected areas or to shift resources elsewhere needs further research. Temporary landscape defragmentation and shifting conservation priorities and investment may offer a flexible alternative to more contested permanent interventions and standard solutions such as networks of protected areas. Identifying and managing conditional biodiversity refugia and developing strategies for temporarily strengthening their connections to the rest of the landscape could help make conservation efforts more resilient to perturbations. Specific circumstances may call for management responses both inside and outside protected areas to reduce risks, provide refuges, or help recolonization (and therefore reorganization).

Second, the evidence for the positive influence of a sustained, meaningful relation to nature for different conservation efforts (from protection to rehabilitation) is still tentative and in need of further research. Crises often expose the potential value of meaningful people–nature relationships, as is evidenced by the increased reliance on visits to parks and forests for recreation and stress relief. They also highlight the fragility and context dependence of positive experiences, and sustaining these during and after a crisis event may require more effort and crisis or trauma tailored mediation. Also, further efforts are required if the effects of these experiences are to persist after the visit, to make them relevant for everyday choices with a bearing on biodiversity conservation. Investigating which conditions enable the development of meaningful relationships with nature in the middle of crisis may help to shape narratives and meanings of biodiversity conservation that remain relevant under changing conditions and to reinforce the support for conservation efforts overall.

Third, rapidly deployable and prenegotiated modes of governance—characterized by clear mandates, pooled resources, shorter decision chains, and intersectoral protocols—can offer critical capacity during disruptions and crises. One avenue of inquiry to advance the mainstreaming of these efforts would be to investigate how biodiversity concerns could be explicitly included in contingency plans and resilience strategies for, for example, food security, education, public health, social equity, and mitigation of extreme weather events.

## Data Availability

There are no new data associated with this article.
